# High-Resolution Spatiotemporal Mapping of Cerebral Metabolism During Middle-Cerebral-Artery Occlusion/Reperfusion Progression: Preliminary Insights

**DOI:** 10.3390/biom15111558

**Published:** 2025-11-06

**Authors:** Zhongcheng Yuan, Minhao Xu, Mingze Lu, Guancheng Wang, Jingyuan Ma, Sitong Ding, Haoan Wu, Yu Zhang, Ming Ma

**Affiliations:** School of Biological Science and Medical Engineering, Southeast University, Nanjing 210096, China; 220232440@seu.edu.cn (Z.Y.);

**Keywords:** ischemia–reperfusion injury, MALDI-MS imaging, microdialysis, spatiotemporal metabolomics, branched-chain amino acids, TCA cycle

## Abstract

Ischemia–reperfusion is a rapidly evolving cascade that involves a variety of metabolic shifts whose precise timing and sequential order are still poorly understood. Clarifying these dynamics is critical for understanding the core injury trajectory of stroke and for refining time-delimited therapeutic interventions. More broadly, continuous in situ monitoring of the middle-cerebral-artery occlusion process at the system level has not yet been achieved. Here, we report the first single-subject high-resolution spatiotemporal resolution metabolic maps of the ultra-early phase of ischemic stroke in a rodent model. Matrix-assisted laser desorption/ionization mass spectrometry (MALDI-MS) imaging mapped a metabolic abnormality area in the ischemic hemisphere that propagates from the striatum to the cortex. Microdialysis probes were then stereotaxically implanted within this metabolic abnormality area, capturing 10,429 metabolites that resolved into 16 temporally distinct trajectories aligned with probe insertion, ischemic injury, and reperfusion injury. Analysis of specific metabolic pathways mainly revealed that the delayed clearance of metabolic waste (urea and tryptamine) during early reperfusion, the transient attenuation of the citrate-to-oxaloacetate buffering gradient within the TCA cycle, and the accumulation of extracellular branched-chain amino acids all play crucial roles in shaping the injury trajectory. Simultaneously, the depletion of cellular repair mechanisms (pyrimidine synthesis) in the early phase of reperfusion also warrants our attention. These findings provide novel insights into the molecular basis and mechanisms of ischemia–reperfusion and offer a comprehensive resource for further investigation.

## 1. Introduction

Stroke is the second leading cause of death and the third leading cause of disability globally [1,2,3]. Of all stroke-related deaths, 62.4% are caused by ischemic events [1]. The high mortality and morbidity rates are due to its defining feature: the “hyperacute phase”. This phase is conventionally defined as the first 0–6 h after onset, but it can sometimes be extended to 24 h [4]. During this period, the core infarct and the surrounding ischemic penumbra evolve rapidly, leading to swift and severe neurological deficits [5,6,7]. The most decisive pathological fluctuations are compressed into the first 12 h [8,9]. Therefore, reconstructing pathway dynamics at a minute-to-hour resolution during the earliest phase of stroke is essential for elucidating its pathogenesis.

Cerebral vessel occlusion ignites a series of cascades that vary significantly across different brain regions and time points, yet they are interconnected [10]. Ischemia–reperfusion injury occurs through reactive oxygen species (ROS) bursts, mitochondrial collapse, and other reactions [11,12,13], eventually converging on three intertwined hubs: energy-metabolic collapse, neuroinflammation, and redox imbalance [14,15]. Cell repair mechanisms and the clearance of metabolic waste are likewise central to neurodegenerative research [16,17,18,19,20]. It is crucial to understand these spatiotemporal differences and how these processes specifically interact at different spatiotemporal levels. Routine imaging techniques, such as CT or MRI, provide “snapshots” of cerebral blood flow, including parameters such as hypoperfusion intensity ratio, T_max_, and relative cerebral blood volume, but they cannot capture the more detailed evolution of these processes [21]. Metabolomics has the potential to reflect these changes from a fundamental perspective [22,23]. Current metabolomic studies mainly rely on blood [24,25,26], cerebrospinal fluid [27,28], or ex vivo tissues [29,30,31,32,33]. The first two are restricted by the blood–brain barrier, while the third cannot achieve longitudinal sampling within the same living subject.

To overcome these limitations, this study first employs matrix-assisted laser desorption/ionization mass spectrometry (MALDI-MS) imaging to delineate spatial metabolite distributions at micrometer resolution and subsequently leverages microdialysis probes for real-time, in situ, continuous sampling; the dialysates are then subjected to high-resolution liquid chromatography quadrupole time-of-flight mass spectrometry (LC-Q-TofMS) metabolomics. The goal is to construct a high spatiotemporal resolution metabolic atlas of ischemic stroke in the ultra-early phase within a single living subject. Additionally, we design an effective algorithm specifically tailored for high-throughput continuous metabolite analysis. This algorithm identifies the unique characteristics of individual metabolites across multiple complex stages, assigning specific temporal fluctuation trends to each metabolite. This facilitates the study of core changes over the 5 h period, including probe insertion, middle-cerebral-artery occlusion (MCAO) surgery, ischemia, and reperfusion.

## 2. Materials and Methods

### 2.1. Ethics and Animal Husbandry

All animal protocols were approved by the Fudan University Laboratory Animal Science Department (approval 2024-HSYY-399, dated 15 May 2024) and conformed to the Chinese Regulations on Laboratory Animals and the 3R principle (Replacement, Reduction, and Refinement). Female SPF-grade Sprague–Dawley rats, 12–14 weeks old and weighing 270–310 g, were purchased from Qinglongshan (Nanjing, China). Upon arrival, animals were housed at 22 ± 2 °C and 50 ± 20% relative humidity under a 12 h light cycle (08:00–20:00) with ad libitum access to food and water. After a 1- to 2-week acclimatization period, during which general health was monitored daily, and any rats showing signs of disease or abnormality were excluded, food was withdrawn 12 h before surgery to minimize anesthesia-related risks and surgical interference. Vaginal smears were collected daily for at least 1–2 weeks before the experiment; only rats displaying consistent diestrus were used.

### 2.2. Transient Middle-Cerebral-Artery Occlusion (tMCAO)

Focal cerebral ischemia was induced by intraluminal occlusion of the right middle cerebral artery. Rats were anesthetized with 1.5–2% isoflurane and maintained at 36.5–37 °C using a rectal probe coupled to a heating pad and lamp. A silicone-coated nylon filament (MSRC40B200PK50; RWD, Nanjing, China, tip Ø 0.38–0.40 mm, length 5–6 mm) was introduced via the right external carotid artery, advanced through the internal carotid artery, and positioned at the MCA origin. After 2 h, the filament was gently withdrawn to restore reperfusion. Sham-operated animals underwent identical procedures without filament insertion.

### 2.3. Tissue Processing and TTC Staining

At the end of reperfusion, rats were euthanized by decapitation under deep isoflurane anesthesia. Brains were snap-frozen in liquid nitrogen, stored at −80 °C, and sectioned coronally (10 µm) at −20 °C on a Leica CM1950 cryostat. Sections were thaw-mounted onto indium–tin-oxide-coated glass slides, vacuum-dried (1 h, RT), and coated with 7 mg mL^−1^ CHCA (Bruker Daltonics, Bremen, Germany) by a 5 V, 400 mA, 2 W mini-humidifier at 35 mL h^−1^. Adjacent 1-mm coronal slices were incubated in 2% TTC in 0.1 M PBS (pH 7.4) at 37 °C for 30 min to delineate infarcted (white) versus viable (red) tissue.

### 2.4. MALDI–MS Imaging and Spatial Data Processing

Spectra were acquired on an UltrafleXtreme MALDI-TOF/TOF (Bruker Daltonics, Bremen, Germany) using a 355 nm Nd:YAG laser in reflectron positive-ion mode (*m*/*z* 50–1000). Each pixel (200 µm × 200 µm) comprised 1000 shots at 1000 Hz. Instrument settings: 2000 Hz laser frequency, 80 ns pulsed ion extraction, 20.00 kV acceleration, 17.90 kV extraction, 5.85 kV lens, 21.15 kV reflectron. Raw data from five sections were imported into SCiLS Lab 2019c (Bruker Daltonics, Bremen, Germany) and R(v4.5.0). Peak picking (±0.180 Da) yielded 285 metabolic features. Hierarchical clustering was performed in SCiLS Lab 2019c (Bruker Daltonics, Bremen, Germany) on the 285 peak-picked features (±0.180 Da) extracted from ~6000 spectra of striatal and hippocampal regions. Prior to clustering, spectra were normalized to their root mean square (RMS) intensity. Agglomerative hierarchical clustering was applied using cosine distance and Ward′s linkage; medium denoising (3-point smoothing) was enabled. The MzRange threshold was set to 3.84 (lower-bound mode), retaining only peaks detected in ≥3.84% of all spectra, and within each ±0.180 Da interval, only the maximum-intensity peak was kept (interval processing mode: Maximum). The resulting spatial clusters were co-registered with adjacent histological sections to annotate grey matter, white matter, and metabolically altered areas. Differential features between ipsilesional and contralesional ROIs were identified and quantified across all sections.

### 2.5. Stereotaxic Surgery

Twenty-four hours before microdialysis, rats were anesthetized with isoflurane (1.5–2%) and maintained on a feedback-controlled heating pad (36.5–37 °C). After fixation in a stereotaxic frame (RWD, Nanjing, China), the scalp was shaved and sterilized with povidone–iodine. Following a midline incision, the periosteum was removed with 3% H_2_O_2_, and a 0.8 mm burr hole (RWD HM1008) was drilled above the right striatum. A guide cannula (CMA 12, shaft length 10.3 mm, outer diameter 0.86 mm, polyurethane; CMA Microdialysis, Sollentuna, Sweden) was implanted at coordinates: +0.7 mm anterior–posterior (AP) and +2.0 mm medial–lateral (ML) relative to bregma, and −4.0 mm dorsal–ventral (DV) from dura, based on previous metabolic-mapping data. Two stainless-steel anchoring screws were inserted adjacent to the craniotomy, and the assembly was secured with dental acrylic. The skin was sutured and treated with 0.5% bupivacaine for post-operative analgesia. A dummy stylet was kept in place to prevent obstruction. Animals were housed individually under a 12 h light/dark cycle with ad libitum food and water.

### 2.6. Microdialysis Sampling and Metabolite Extraction

On the day of the experiment, the dummy stylet was replaced with a concentric microdialysis probe (CMA 12, 4 mm membrane, 20 kDa cutoff; CMA Microdialysis, Sweden). The probe was perfused with artificial cerebrospinal fluid (aCSF: 145.0 mM NaCl, 2.7 mM KCl, 1.0 mM MgCl_2_, 1.2 mM CaCl_2_, 0.45 mM NaH_2_PO_4_, 2.33 mM Na_2_HPO_4_; pH 7.4; Sigma-Aldrich, St. Louis, MO, USA) at 2.33 μL min^−1^ (TS-2A, lange, Longer Precision Pump, Nanjing, China). After a 60 min stabilization period, dialysates were collected every 15 min into chilled polypropylene vials containing 4 μL 0.1 M acetic acid to suppress auto-oxidation (final collection volume 35 μL). Four baseline samples were obtained prior to middle cerebral artery occlusion (MCAO). All samples were snap-frozen in liquid N_2_ and stored at −80 °C until analysis.

Microdialysate (35 μL) was deproteinized with 140 μL ice-cold acetonitrile/methanol (1:1, *v*/*v*). After vortexing (30 s) and sonication on ice (10 min), the mixture was incubated at −20 °C for 1 h to enhance precipitation. Samples were centrifuged at 12,000× *g* for 15 min at 4 °C. The supernatant was dried in a vacuum concentrator and reconstituted in 100 μL acetonitrile/water (1:1, *v*/*v*). Following a second centrifugation (12,000× *g*, 15 min, 4 °C), the final supernatant was transferred to LC-MS vials.

### 2.7. LC-MS/MS and Data Processing

Metabolites were separated on an Agilent 1290 Infinity II UHPLC coupled to an Agilent 6546 Q-TOF mass spectrometer. Chromatography was performed on an ACQUITY UPLC HSS T3 column (2.1 × 100 mm, 1.8 μm; Waters, Milford, MA, USA) maintained at 40 °C. The mobile phase consisted of (A) 0.1% formic acid in water and (B) 0.1% formic acid in acetonitrile delivered at 0.4 mL min^−1^. The gradient began at 5% B (1 min), increased linearly to 95% B over 15 min, held for 3 min, returned to 5% B within 0.5 min, and re-equilibrated for 3.5 min. The injection volume was 5 μL. The Q-TOF was operated in full-scan mode (*m*/*z* 50–1300) at 2 GHz extended dynamic range. Electrospray ionization was employed in both positive (capillary +3.5 kV) and negative (−3.0 kV) modes. Source parameters were as follows: drying gas, 200 °C at 12 L min^−1^; nebulizer, 35 psig. Data were acquired at 5 spectra s^−1^. Auto MS/MS experiments were conducted with stepped collision energies of 10, 20, and 40 eV. Continuous infusion of reference masses (*m*/*z* 121.0509 and 922.0098 in positive mode; 119.0363 and 966.0007 in negative mode) via the AJS-ESI source ensured mass accuracy <2 ppm.

Raw LC-MS files (.d) were converted to mzML and processed in MS-DIAL (v5.5.250530) and R (v4.5.0). Peaks with intensity >500 counts in both positive and negative modes were aligned across 105 sequential samples. Peak lists were normalized by total ion current, batch-corrected using LOESS regression, and gap-filled. A total of 10 529 annotated features were retained. Temporal dynamics were analyzed using the Mfuzz package (v2.68.0). After determining the optimal fuzzifier parameter m, features were partitioned into 16 clusters based on their time-resolved profiles. Membership values ${\mathrm{lg}u}_{\mathrm{ik}}$ were used as soft labels for subsequent hierarchical clustering (HCA) performed with Ward’s linkage and Euclidean distance. n represents the total number of samples, c is the number of clusters, and m is the fuzzifier. $u_{ik}$ denotes the membership degree of sample i to cluster k, and x and v are the feature vectors of the sample and cluster centers.

$$\mathrm{XB}((1)m)=(\sum_{i=1}^{n}\sum_{k=1}^{c}u_{\mathrm{ik}}^{m}\|x_{i}-v_{k}{\|}^{2})/(n\cdot \mathrm{mi}n_{k\neq l}\|v_{k}-v_{l}{\|}^{2})$$

$$u_{\mathrm{ik}}={\left[\sum_{j=1}^{c}\left(\frac{\left\|x_{i}-v_{k}\right\|}{\left\|x_{i}{-v}_{j}\right\|}\right)\right]}^{-\frac{2}{m-1}}$$

### 2.8. Enrichment and Pathway Analysis

Metabolites displaying abrupt fluctuations at critical time points (5 clusters corresponding to injury phases) were subjected to enrichment analysis in MetaboAnalyst 6.0 (https://www.metaboanalyst.ca, accessed on 15 May 2025) using the “MS peaks to pathways” workflow for Rattus norvegicus. Pathways with Fisher’s exact test *p* < 0.01 were curated against the HMDB and visualized. Temporal trajectories of key metabolites were plotted as fold-change relative to SD Energy.

### 2.9. PLS-R and LASSO Regression

PLS-R and LASSO regression analyses were performed using R (v4.5.0) and MetaboAnalyst 6.0. We employed i-PCA to expand the 10,529 metabolites to determine whether the samples were in the ischemic or non-ischemic state. The metabolites, annotated with their respective types and aligned, were expanded in a six-dimensional temporal sequence to obtain the PLS-R results. For the first principal component, the top ten metabolites with the highest VIP values were subjected to LASSO regression, while for the second and third principal components, the top ten metabolites with the highest VIP values were analyzed using sPLS-DA. ROC curves were subsequently constructed for each metabolite combination in their respective spaces.

## 3. Results

### 3.1. Spatial Metabolic Maps

**Figure 1:**
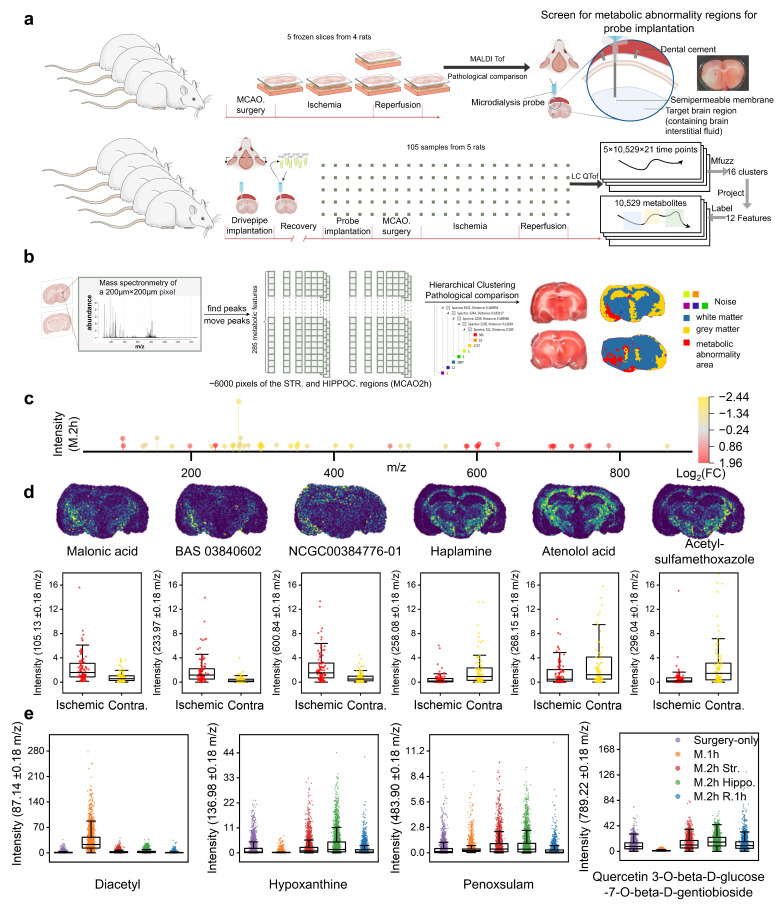
Spatial metabolic analysis in MCAO/R rat brain regions: (**a**) Upper panel: Schematic diagram of the sampling scheme for metabolic and pathological information in different brain regions at various MCAO/R stages in rats, and the metabolic abnormality area determined by MALDI TOF following microdialysis probe insertion. Lower panel: Workflow for continuous sampling in the striatal region of the rat brain using microdialysis to label MCAO/R characteristics of metabolites. (**b**) Peak extraction from MALDI results of ischemic striatal and hippocampal brain regions at 2 h of ischemia, followed by hierarchical clustering to identify metabolic abnormality area (red area) in comparison with TTC results and anatomical structures. (**c**) Differential metabolic features between the metabolic abnormality area and the contralateral symmetric area in the brain at 2 h of MCAO (|log_2_FC| > 1). (**d**) Spatial metabolic maps of representative differential expression metabolites (Malonic acid, BAS 03840602, NCG000384776-01, haplamine, atenolol acid, and acetylsulfamethoxazole) and the box plots of expression levels for each pixel of these metabolites in the metabolic abnormality area and contralateral symmetric area. (**e**) Expression levels for each pixel across the whole brain slices of four representative metabolic features (from left to right, purple indicates the only surgery group, orange indicates the MCAO 1 h group, red and green indicate the striatum and hippocampus slices of the MCAO 2 h group, and blue indicates the MCAO 2 h reperfusion 1 h group).

To construct metabolic maps of different brain regions during the metabolic development process, brain cryosections were performed on rats in the only surgery group (MCAO 0 h), MCAO 1 h, MCAO 2 h, and MCAO 2 h reperfusion 1 h groups. The MCAO 2 h group had double sections taken from the striatum and hippocampus, while the other groups had single sections taken from the striatum (Figure 1a). Subsequently, matrix spraying and MALDI were conducted on the five selected sections, generating approximately 3000 regions of 200 μm × 200 μm each with metabolic information. Metabolic maps were scanned in the range of 50 to 1000 *m*/*z*, and ultimately, 285 metabolic features were identified, and their peaks were determined.

As shown in Figure 1b, unsupervised hierarchical clustering was performed on each pixel region of the striatal and hippocampal sections from the MCAO 2 h rat, resulting in three main cluster regions and five additional regions treated as noise. Compared with the anatomical brain region results, the metabolic abnormality area was clearly demarcated from the normal gray/white matter areas. The metabolic abnormality area was localized to the extended region from the striatum to the cortex in the ischemic hemisphere. Cross-sectional TTC staining demonstrated that the ischemic lesion originated in the striatum and subsequently expanded to the hippocampus and cortex (see Figure S1). Therefore, the metabolic abnormality area was confirmed as the stroke-specific region in the ischemic hemisphere.

Differential analysis was conducted on the 285 metabolic features in the metabolic abnormality area and the contralateral symmetric area. Figure 1c presents the 50 differential metabolic features with |log_2_ FC| > 1. Among them, 21 metabolic features were upregulated in the metabolic abnormality area of the ischemic hemisphere (Table S1), while 29 metabolic features were downregulated (Table S2). The spatial distribution of representative metabolites that were upregulated and downregulated in the metabolic abnormality area of the ischemic hemisphere under the MCAO 2 h condition, as well as the expression levels of each pixel, are demonstrated (Figure 1d). We have demonstrated the following metabolites: Malonic acid, BAS 03840602, NCG000384776-01, Haplamine, atenolol acid, and acetylsulfamethoxazole. Additionally, Figure 1e shows the spatiotemporal differential distribution of four metabolites across the whole sections in the only surgery group, MCAO 1 h, and MCAO 2 h reperfusion 1 h groups (Figures S2 and S3). Diacetyl was significantly downregulated in the MCAO 1 h group; in contrast, hypoxanthine and quercetin 3-O-beta-D-glucose-7-O-beta-D-gentiobioside were upregulated at this time point, while penoxsulam remained relatively stable.

### 3.2. Temporal Metabolic Maps with Labeling

**Figure 2:**
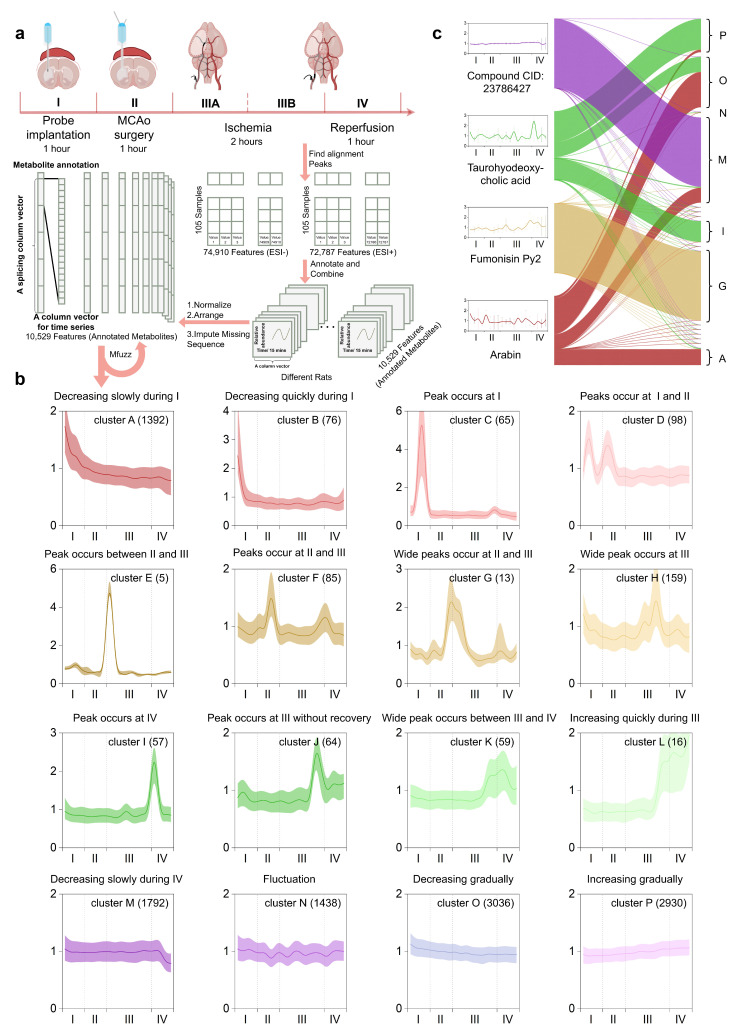
MFuzz-based metabolite feature annotation: (**a**) The entire procedure was operationally segmented into five phases: Phase I (probe implantation), Phase II (MCAO filament insertion), Phase IIIA (early ischemia), Phase IIIB (late ischemia), and Phase IV (reperfusion). Metabolic fingerprints of each time-stamped microdialysate were pre-processed, binarized, and stacked into high-dimensional column vectors for optimal fuzzifier estimation and Mfuzz clustering. (**b**) 16 Mfuzz-extracted temporal trajectories were annotated according to their characteristic dynamics across the defined metabolic phases. (**c**) Mfuzz-derived 16 metabolic labels were mapped onto tag-connecting plots for four representative metabolites.

After determining the probe insertion site via spatial metabolic maps (bregma: AP +0.7 mm, ML +2.0 mm, DV −4.0 mm), we implanted microdialysis guide cannulae 24 h before MCAO/R and advanced the probes into the ventral striatum 1 h before occlusion (Figure 1b). A total of 105 serial perfusate samples (21 samples per rat) were collected across five animals, spanning a 1 h baseline, 1 h MCAO surgery, 2 h ischemia, and 1 h reperfusion (Figure 1a). LC-Q-TofMS analysis yielded 74,910 (ESI−) and 72,787 (ESI+) ion features; after quality filtering, gap-filling, and annotation, 10,529 non-redundant metabolic signatures were retained and arranged as labeled longitudinal vectors (Figure 2a).

The fuzzifier m and cluster number (c = 16) were optimized to maximize the ratio of intra-cluster tightness to inter-cluster separation while preserving the full stroke trajectory, in order to identify the dynamic patterns of significant metabolic events during MCAO/R (Figure 2b). Each of the 16 clusters, therefore, represents a distinct kinetic signature across baseline (I), occlusion (II–III), and reperfusion (IV) stages. Clusters A and B exhibited gradual and rapid declines, respectively, during period I. Cluster C showed a sharp increase followed by a decrease during period I. Cluster D had peaks during both periods I and II. Cluster E had a peak at the boundary between periods II and III. Clusters F and G both had peaks during periods II and III. Cluster H had a broad peak during period III. Cluster I had a peak at the boundary between periods III and IV. Cluster J had a peak at the end of period III and did not fully recover afterward. Cluster K had a broad peak at the boundary between periods III and IV. Cluster L showed a rapid increase during period IV. Cluster M had a gradual decline during period IV. Cluster N exhibited continuous fluctuations throughout all periods. Clusters O and P had gradual declines and increases, respectively, throughout all periods. Based on the characteristics of these 16 temporal clustering patterns, we manually annotated them.

Due to the soft-clustering nature of MFuzz, a single metabolic feature can simultaneously exhibit high membership in multiple stroke-evolution trajectories (Figure 2c), which mirrors the biological reality that one metabolite may engage in several core pathological processes. We therefore leveraged the final-iteration membership coefficient and assigned it as the quantitative label for every metabolite. Under this framework, the distance from the cluster centroid inversely correlates with membership strength, while a higher fuzzifier m broadens the membership distribution and increases ambiguity. Four corresponding examples shown in Figure 2c illustrate the assigning principle: CID:23786427 is uniquely associated with the trajectory “gradually decreasing during IV”; Fumonisin Py2 is uniquely associated with the trajectory “wide peaks occur at II and III”; Taurohyodeoxycholic acid is associated with the trajectories “increasing gradually” “decreasing gradually” and “peak occurring at IV”; Arabin is associated with three declining signatures “decreasing gradually” “decreasing slowly during IV” and “decreasing slowly during I”.

### 3.3. Metabolite Pathway Analysis

**Figure 3:**
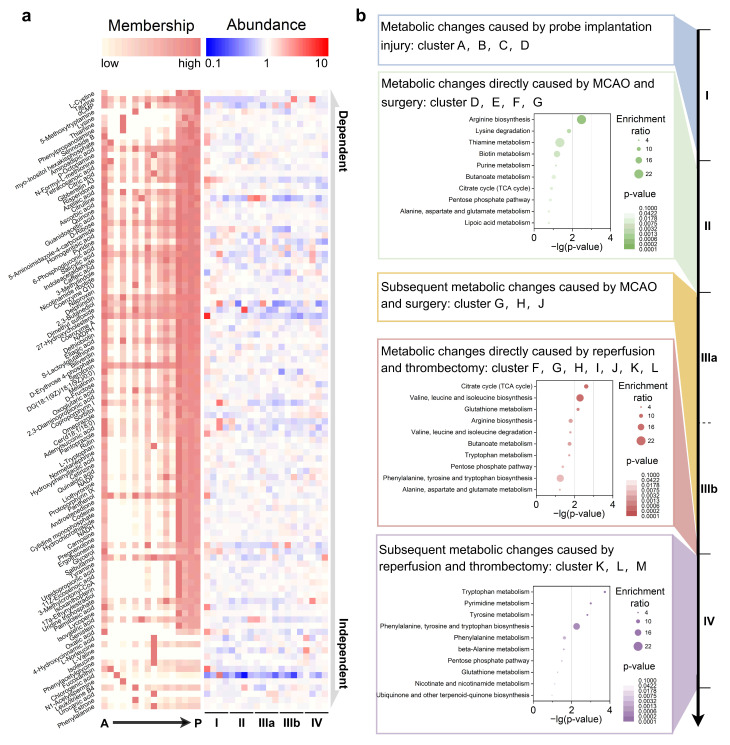
Metabolic atlas of MCAO/R stages and corresponding core pathways: (**a**) Metabolites with membership values > 0.5 in clusters A–P are shown. Higher positioning indicates a stronger association with Clusters M, N, O, and P, while lower positioning shows more independence and similarity to MCAO/R labels. The right side displays original relative abundance in MCAO/R stages. (**b**) Projection of the updated MFuzz-derived kinetic signatures onto metabolites during the MCAO/R period for KEGG enrichment analysis. KEGG enrichment of metabolites assigned to Clusters D–G, whose levels fluctuate significantly across the II → IIIA transition. KEGG enrichment of metabolites assigned to Clusters F–L, whose levels fluctuate significantly across the IIIB → IV transition. KEGG enrichment of metabolites assigned to Clusters K–M, whose levels remain significantly altered throughout Phase IV.

It was observed that Taurohyodeoxycholic acid had a peak in the early stage of period IV but exhibited a high weight in the P cluster (Figure 2c). This seemingly incongruous feature assignment to certain metabolites is due to the particularity of the algorithm. By using the results of MFuzz to perform hierarchical cluster analysis (HCA) on metabolites with KEGG annotations based on their membership values (Figure S5), it was found that the algorithm exhibits a “drowning effect”: some features with significant trends are easily overshadowed by dominant clusters. For example, as shown in Figure 3a, the majority of metabolites have high membership values in Clusters M, N, O, and P. Therefore, improvements were made by defining the highest membership cluster outside of M, N, O, and P for a given metabolite as its primary characteristic.

Metabolites of significant biological relevance in the Metlin and KEGG databases were analyzed, and those with membership values higher than 0.5 in Clusters A to L were selected for presentation (Figure 3a). The higher the metabolites are positioned, the more strongly they are attracted to Clusters M, N, O, and P. In contrast, the lower ones are relatively more independent and show higher similarity to the labels we screened for MCAO/R. On the right side, their original relative abundance in each stage of MCAO/R is displayed. Each of the 12 updated MFuzz-derived kinetic signatures was aligned with three distinct insults: probe implantation, ischemic occlusion, and reperfusion/thrombectomy, resulting in a refined metabolic roadmap of stroke progression.

As shown in Figure S6, acute stereotactic probe insertion triggered a metabolically diffuse response. 47 KEGG-annotated metabolites (Clusters A–D) showed no significant enrichment in any canonical pathway, indicating that micro-trauma initiates a nonspecific biochemical cascade rather than a coordinated metabolic program. Immediate vessel occlusion and surgical stress rapidly rewired nitrogen metabolism. 102 metabolites (Clusters D–G) became selectively enriched in arginine biosynthesis (Figure 3b), consistent with an urgent nitric oxide–driven reprogramming aimed at countering operative ischemia. Persistent hypoperfusion maintained metabolic disarray. As shown in Figure S7, 31 metabolites (Clusters G, H, and J) exhibited no dominant pathway signature, suggesting that the metabolic network remains uncoupled during ongoing ischemia and has not yet transitioned to a coherent adaptive state.

Re-establishment of cerebral blood flow elicited a burst of oxidative and anabolic activity. 83 metabolites (Clusters F–L) converged on the citrate cycle, valine–leucine–isoleucine biosynthesis, and glutathione metabolism (Figure 3b), underscoring simultaneous reactive oxygen species detoxification and ATP resynthesis as hallmarks of acute reperfusion. Post-reperfusion remodeling was dominated by aromatic amino acid and nucleotide anabolism. 245 metabolites (clusters K–M) were significantly enriched in tryptophan, pyrimidine, and tyrosine metabolism (Figure 3b), as well as the integrated phenylalanine, tyrosine, and tryptophan biosynthetic axis. Collapsing these overlapping pathways revealed two dominant signatures: phenylalanine, tyrosine, tryptophan biosynthesis, and pyrimidine metabolism. These collectively define the biochemical chronology of ischemic injury initiation, propagation, and recovery, thereby providing mechanistic anchors for therapeutic targeting.

### 3.4. MCAO/R Metabolic Reprogramming

**Figure 4:**
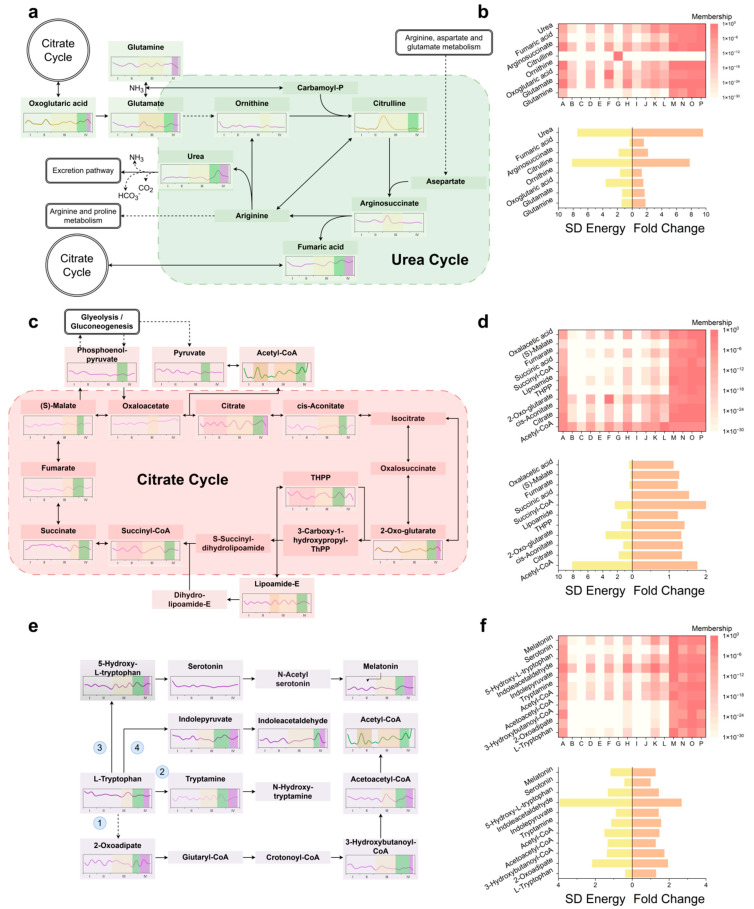
Dominant metabolic pathways and their characteristics during MCAO/R progression: (**a**) Expansion of metabolite profiles within the KEGG-defined “Arginine biosynthesis” pathway implicated in direct ischemic injury. (**b**) Top: cluster-label mapping for arginine-biosynthesis metabolites. Bottom: SD Energy (global temporal fluctuation) and key-time fold change (signature-period fold change). (**c**) Expansion of metabolite profiles within the KEGG-defined “Citarate circle” pathway implicated in reperfusion-induced acute injury. (**d**) Top: cluster-label mapping for Citarate circle metabolites. Bottom: SD energy and key-time fold change. (**e**) Expansion of metabolite profiles within the KEGG-defined “Tryptophan metabolism” pathway implicated in sustained reperfusion injury. The numbers 1–4 correspond to the four consumption pathways in tyrosine-centered metabolism. (**f**) Top: cluster-label mapping for tryptophan-metabolic metabolites. Bottom: SD energy and key-time fold change.

During ischemic Phase III (Figure 4a), glutamate levels spiked within minutes (fold change greater than 2) and then rapidly fell below baseline. In contrast, glutamine exhibited two smaller maxima in Phases IIIA and IIIB, with fold changes of approximately 1.6 and 1.7, respectively. Citrulline, which is committed to the urea cycle, displayed a broad plateau with a fold change greater than 5 and a duration of over 1 h (Figure 4b). Argininosuccinate modestly increased in Phase IIIA (fold change approximately 1.5), while fumarate rose in Phase IIIB (fold change approximately 1.4). Upon reperfusion, urea surged (fold change greater than 9), indicating rapid nitrogen clearance (Figure 4b).

In the early reperfusion period, the tricarboxylic acid (TCA) cycle showed distinct changes (Figure 4c). Acetyl-CoA developed biphasic peaks during ischemia and reperfusion, with the reperfusion peak having a fold change greater than 4. Citrate levels fell to less than 0.5 times the baseline during mid-ischemia but rebounded synchronously with acetyl-CoA. A stepwise attenuation was observed from the entry (acetyl-CoA) to the exit (oxaloacetate) of the TCA cycle (Figure 4d). The branched-chain amino acids isoleucine (fold change greater than 3) and valine (fold change greater than 4) also surged (Figure S8). Within glutathione metabolism (Figure S9), GSSG increased to 1.5 times the baseline, NADPH to 1.8 times, while NADP^+^ decreased by 45%. γ-glutamyl cycle intermediates 5-oxoproline and L-cysteinyl-glycine peaked at 1.6 times and 1.4 times the baseline, respectively, and the constituent amino acids (glutamate, glycine, and cysteine) all increased, with glycine showing a delayed rise. Polyamine levels remained stable throughout.

Tryptophan, an aromatic amino acid hub, peaked early in reperfusion and then stabilized (Figure 4e,f). Its alternative catabolic route to fumarate (route 1) was activated, with downstream metabolites 2-oxoadipate, 3-hydroxybutanoyl-CoA, and acetoacetyl-CoA reaching marked maxima during reperfusion. The decarboxylase (DDC) route (route 2) drove a transient rise in tryptamine, which then declined. 5-Hydroxytryptophan (5-HTP, route 3) initially fell and then rebounded, while melatonin showed the opposite trend; serotonin remained within baseline limits post-reperfusion. Indole-3-pyruvate (route 4) dipped before re-ascending, whereas indole-3-acetaldehyde rose and then subsided. In tyrosine-centered metabolism (Figure S10), route 1 metabolites remained stable during reperfusion. Tyramine (route 2) initially dropped and then climbed, while homogentisic acid rose continuously. Phenylpyruvate (route 3) displayed a sharp peak during the reperfusion phase. Thyroxine (route 4) increased (fold change 1.33) and then fell (fold change 0.60) before stabilizing. Within pyrimidine metabolism (Figure S11), UTP spiked transiently (fold change greater than 3) and then returned to baseline.

### 3.5. StrokeAssure and StrokeProgressionTracker Models

To better leverage metabolic markers for predicting the stroke stage of patients and thereby provide diagnostic and therapeutic strategies, we initially employed i-PCA to expand the 10,529-dimensional metabolic features around the ischemic time points (8 and 9) (Figure S13). The results from PC1 to PC4 show that the clustering of different rats did not separate well, with all rats clustering into five categories. However, the expansion of PC5 achieved good separation between the ischemic state (red) and the non-ischemic state (blue).

**Figure 5:**
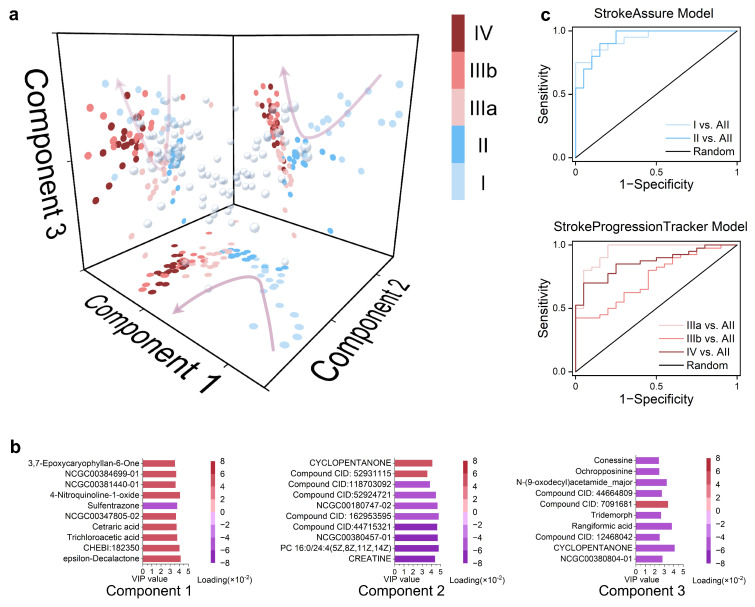
StrokeAssure and StrokeProgressionTracker models for screening stroke biomarkers using PLS-R and Lasso Penalt: (**a**) Scatter plot obtained from the interaction of the first three components derived from PLS-R performed on 10,529 metabolites based on the stage classification of all samples, with colors indicating the stage of the samples. (**b**) The top 10 metabolites with the highest VIP values for each Component obtained in (**a**), with colors representing the loadings for the components. (**c**) ROC curves for predicting the pre-stroke stage using the five metabolites in Component 1 and the six metabolites in Components 2 and 3, respectively, after LASSO regression was applied to the top ten metabolites with the highest VIP values for each Component obtained in (**a**).

Subsequently, using the 10,529-dimensional metabolic features as the dependent variable, the different stages were expanded into six dimensions (Figure S12), and PLS-R was performed, with the first three components selected for analysis (Figure 5a). It was observed that in the pre-ischemic stages (I and II), Component 1 showed good expansion and was negatively correlated with stroke progression time. In the post-ischemic stages (IIIA, IIIB, and IV), Components 2, 3 exhibited better expansion, with the former being negatively correlated with stroke progression time and the latter positively correlated. The top ten metabolites with the highest VIP contributions and their loadings for each component are also presented (Figure 5b).

The top ten metabolites with the highest VIP contributions were directly used, and these metabolites could distinguish each stage with 100% accuracy (Figure S13). Therefore, further reduction in the metabolite panel size is needed. The L1 penalty was introduced to Components 1, 2, and 3 to identify the minimal biomarker signature. The former task can be understood as conventional LASSO regression, which resulted in metabolite combination 1: CHEBI:182350, trichloroacetic acid, cetraric acid, NCGC00347805-02, and 4-nitroquinoline-1-oxide. Based on this combination, the StrokeAssure Model was established. The latter task involved a three-class sPLS-DA, yielding metabolite combination 2: CREATINE, PC 16:0/24:4(5Z,8Z,11Z,14Z), NCGC00380457-01, cyclopentanone, NCGC00380804-01, and 8-acetyl-5,7-dimethoxy-2,2-dimethylchromene. Based on this combination, the StrokeProgressionTracker model was established. Their respective ROC curves are shown in Figure 5c.

## 4. Discussion

The metabolomics studies of stroke and the discovery of IS biomarkers face two main challenges [34]. First, the presence of the blood–brain barrier prevents large molecules from being released from the brain into the blood. Second, there is the high heterogeneity of IS. In previous metabolomics studies of stroke using MALDI, the individual differences were often ignored because the slices used were from different mice, and the modeling differences usually exacerbated this drawback [35,36,37]. Meanwhile, past metabolomics studies typically only indicated whether a certain metabolite increased or decreased during ischemia. Specifically, lysine, phenylalanine, methionine, tryptophan, leucine, lactate, ethanolamine, alanine, isoleucine, valine, tyrosine, 3-hydroxybutyrate (3-HB), glycerol, γ-aminobutyric acid (GABA), and glycine were found to increase, while aspartate, taurine, serine, N-acetylaspartate (NAA), and glutathione (GSH) were found to decrease [38,39]. This is a simplistic interpretation of past metabolomics findings, which is obviously insufficient to explain the complex changes in stroke.

On the other hand, conventional K-means clustering, with its inherent hard-partitioning constraint, obliges each metabolite to affiliate with a single cluster [40,41]. While this approach adequately captures dominant trends in low-complexity datasets with sparse temporal resolution, it fails when confronted with the pleiotropic nature of metabolism—where a single metabolite frequently participates in multiple, temporally distinct pathways [42]. To overcome this limitation, we employed Mfuzz, a soft-clustering algorithm extensively validated in transcriptomic and proteomic arenas. By iteratively assigning membership fuzzifier (m) coefficients between 0 and 1 [43,44,45]. Mfuzz enables metabolites to simultaneously contribute to several clusters while minimizing the overall cluster number. This strategy markedly enhances the interpretability of high-dimensional, time-resolved metabolic networks and more accurately reflects the biological reality underlying complex ischemia–reperfusion trajectories.

This study is the first to combine metabolite-guided positioning of the ischemic region with high-temporal-resolution microdialysis–LC–MS, enabling continuous, in situ, minute-scale tracking of the brain interstitial-fluid metabolome throughout the entire ischemia–reperfusion cycle, thereby overcoming the static snapshots inherent to blood, cerebrospinal fluid, or post-mortem tissue [46,47,48,49,50].

Although a few studies diverge from our findings, for example, Dunbing Huang et al. reported downregulation of arginine and proline metabolism in the feces of ischemic mice [51], while Rashad S et al. observed upregulation of pyrimidine metabolism in ischemic mouse brain [52] (we demonstrate that this pathway is transiently elevated and then declines during early reperfusion)—these discrepancies can be attributed to differences in sampling sites and pathological time windows. Conversely, our data corroborate previous reports by Wang X et al., who documented increased serum levels of leucine-isoleucine, proline, threonine, glutamate, and arginine pathways [52], and by Sidorov EV et al., who identified elevations in ketone bodies, branched-chain amino acids, energy metabolites, and inflammatory compounds [53]. Beyond refining or corroborating the aforementioned views, we provide more direct evidence, finer temporal resolution, and a systematic examination of the accompanying alterations in neighboring metabolic nodes.

The resulting dynamic flux maps reveal three metabolic processes hitherto overlooked that reshape current concepts of post-stroke injury. First, the temporal-window toxicity of metabolic waste—exemplified by urea [54,55] and tryptamine [56,57]—does not arise during ischemia itself but becomes apparent during early reperfusion because of delayed clearance (Figure 4a,e). The resulting osmotic load possibly represents a novel metabolic–osmotic coupling mechanism for cytotoxic edema [58,59]. Second, the TCA cycle displays a stepwise buffering capacity in which the transient decoupling of the citrate/acetyl-CoA gradient acts as a graded brake that attenuates the reactive oxygen species burst when oxygen suddenly re-enters the tissue [60], thus providing a molecular chronometer for the minute-level protection observed in ischemic preconditioning (Figure 4c,d). Third, the accumulation of extracellular branched-chain amino acids and their α-ketoacids—resulting from PPM1K-BCKDH axis inhibition—suppresses the glutamate–glutamine cycle and diminishes GABA synthesis [61,62], thereby bridging classical excitotoxicity to a global nitrogen–carbon network reconfiguration.

During the ischemic phase, glutamate erupts within seconds, over-activating acid-sensing ion channels and amplifying toxicity [23], while astrocytes immediately recruit glutamine synthetase to generate a transient glutamine peak that marks phase-IIIA metabolic compensation [63]. As ATP partially recovers in phase IIIB, glutamine rises again, indicating renewed glutamate–glutamine shuttling in surviving cells (Figure 4a), yet the synchronous citrulline bulge and fumarate accumulation expose a mitochondrial bottleneck that constrains urea-cycle-mediated ammonia detoxification [58,59,64].

During the early phase of reperfusion, acetyl-CoA exhibits a biphasic profile: an ischemic surge driven by PDH-mediated pyruvate shunting and a higher second peak during early reperfusion caused by simultaneous influx of fatty acids and pyruvate before full TCA flux recovery, with the flattening citrate/acetyl-CoA gradient serving as an intrinsic redox damper [65,66,67] (Figure 4c,d). Oxidative stress is confirmed by GSSG accumulation and NADPH/NADP^+^ imbalance(Figure S9); transient activation of the pentose-phosphate pathway and malic enzyme supplies fleeting reducing power, while elevated γ-glutamyl cycle intermediates signal acute GSH demand [68,69]. Glycine, rising belatedly alongside residual glutamate, synergistically over-activates NMDA receptors to deliver an “oxidative–excitatory” dual hit [70,71], and despite increased cysteine availability, net NADP^+^ depletion ultimately exhausts antioxidant capacity [72].

Meanwhile, breakdown of the blood–brain barrier floods the interstitium with aromatic amino acids [73,74,75], and phenylalanine and tyrosine are diverted through the phenylalanine–tyrosine–fumarate bypass that yields auxiliary energy at the cost of lactate accumulation (Figure 4e and Figure S10), whereas tryptophan is rerouted by DDC to tryptamine [76,77], which—via AhR signaling—may initially exert anti-inflammatory effects but later turns toxic [78,79]. The biphasic serotonin–melatonin trajectory suggests compensation followed by depletion, and increased conversion of indole-3-pyruvate to indole-3-acetaldehyde marks a shift from protective to deleterious tryptophan metabolism [80,81].

Moreover, thyroxine rises and then falls in concert with MCT8/OATP1C1–DIO2/3 expression [82], indicating a narrow time window in which thyroid hormone acts as a mitochondrial uncoupler and ROS amplifier that can still accelerate neuronal death [83,84]. Early reperfusion UTP elevation signals initiation of DNA repair (Figure S10) [85], while subsequent suppression of DHODH and related enzymes causes a sharp synthetic decline [86], revealing long-range mitochondrial inhibition of nucleotide metabolism. Net consumption of amino acids and nucleotides further indicates that energetic collapse and futile repair jointly drive delayed cell death (Figure 4e, Figures S10 and S11).

Compared with other stroke models [87,88,89], our study with 100 samples provided a more interpretable metabolite combination for distinguishing pre- and post-stroke periods and different stages after stroke through in situ longitudinal sampling. The StrokeAssure model can preliminarily screen metabolite combinations for monitoring stroke occurrence or aiding clinical decisions, needing further validation in blood or cerebrospinal fluid. Given the inherent differences between rodent and human models, the time course of stroke progression may differ by several folds [90]; therefore, direct temporal alignment should be established by comparing readily accessible human compartments—namely, blood and cerebrospinal fluid—with the corresponding rodent samples. The StrokeProgressionTracker model can help determine stroke duration and assess spontaneous reperfusion. There have been relevant experiments focusing on the detection of neuromodulators in vivo with high spatiotemporal resolution, or conducting intracellular studies on specific metabolites such as glutamate [22,23]. Although microdialysis is confined to the extracellular space, future integration of single-cell mass spectrometry with ^13^C tracing will clarify the cell-type-specific roles in metabolic reprogramming. Additionally, targeted interventions in branched-chain amino acid oxidation, urea handling, and tryptophan catabolism will validate their therapeutic potential.

This study has several limitations. The demand for high temporal density and high-precision metabolomics inevitably increases economic cost, forcing us to reduce sample size—specifically, one rat per time point for spatial profiling and longitudinal sampling of only five rats for temporal profiling. Consequently, stricter requirements are imposed on both sample selection and algorithm robustness. Moreover, depicting stroke progression solely from a metabolic perspective is inherently incomplete; integrating spatiotemporal transcriptomics, post-translational modifications, and genomic analyses is essential to create a more comprehensive view. Fortunately, many studies have already delved deeply into individual metabolic pathways, and their findings corroborate several of our observations [91,92]. Across our dataset and >70% of prior mouse IS reports, 20 consistent metabolites (e.g., Lys, Phe, Met, Trp, Leu, Lac, Ser, NAA, GSH) and four core pathways (arginine biosynthesis; Ala-Asp-Glu metabolism; aminoacyl-tRNA biosynthesis; and citrate cycle) align with human plasma/CSF signatures [38]. Five circulating metabolites (Pro, Ser, lysoPC(16:0), UA, and Glu) and seven corresponding pathways are shared between rodent models and clinical cohorts, underscoring their translational value for early diagnosis and therapeutic targeting of ischemic stroke.

## 5. Conclusions

In summary, this study elucidated the intricate metabolic changes in the brain following MCAO/R, which encompassed alterations in various metabolic pathways, including the delayed clearance of metabolic waste (urea and tryptamine) during early reperfusion, the transient decoupling of the citrate/acetyl-CoA gradient in the TCA cycle, and the accumulation of extracellular branched-chain amino acids (BCAAs). These metabolic shifts are likely to play pivotal roles in modulating the injury trajectory by affecting energy metabolism, neurotransmitter signaling, and cellular repair mechanisms. Our findings provide novel insights into the molecular basis and mechanisms of ischemia–reperfusion injury and offer a comprehensive resource for further investigation into therapeutic interventions targeting these critical metabolic pathways.

## Data Availability

Relevant code has been deposited at GitHub and is publicly available at https://github.com/yuanzhongcheng409/spatiotemporal-mapping-of-cerebral-metabolism (accessed on 4 September 2025) as of the date of publication. Any additional information required to reanalyze the data reported in this paper is available from the lead contact upon request.

## Supplementary Materials

The following supporting information can be downloaded at: https://www.mdpi.com/article/10.3390/biom15111558/s1, Figure S1: TTC-stained brain slices from different groups; Figure S2: MALDI results from coronal sections of MCAo 2 h; Figure S3: MALDI results from coronal sections of MCAo 2 h reperfusion 1 h, the only surgery group and MCAo 1 h; Figure S4: The complete surgical procedure and corresponding stages of injury; Figure S5: Hierarchical cluster analysis (HCA) of metabolites labeled with KEGG tags (A–P); Figure S6: KEGG pathway analysis of brain injury related to metabolites from stereotactic probe insertion; Figure S7: KEGG pathway analysis of brain injury related to metabolites associated with subsequent metabolic changes caused by MCAo and surgery; Figure S8: Valine, leucine, and isoleucine biosynthesis pathway metabolites analysis; Figure S9: Glutathione metabolism pathway metabolites analysis; Figure S10: Tyrosine metabolism pathway metabolites analysis; Figure S11: Pyrimidine metabolism pathway metabolites analysis; Figure S12: Parameter settings for each dimension in PLS-R (partial least squares regression); Figure S13: Unfolding of 10,529-dimensional metabolic features using i-PCA (incremental principal component analysis); Figure S14: ROC curves for stage determination using the top 10 metabolites with the highest VIP (variable importance in projection) values for each component; Table S1: Metabolic features upregulated in the metabolic abnormality area of the ischemic hemisphere; Table S2: Metabolic features downregulated in the metabolic abnormality area of the ischemic hemisphere.
