# Supplementary material for: High-Resolution Spatiotemporal Mapping of Cerebral Metabolism During Middle-Cerebral-Artery Occlusion/Reperfusion Progression: Preliminary Insights
Source: Biomolecules. 2025 Nov 6;15(11):1558. doi: 10.3390/biom15111558 (PMC12650308; doi:10.3390/biom15111558)
Supplement: Supplementary file 1 [file biomolecules-15-01558-s001.zip › biomolecules-3904293-supplementary.pdf]

High-resolution spatiotemporal mapping of cerebral metabolism during middle cerebral artery ischemia/reperfusion progression-Supplementary information

Zhongcheng Yuan[1,#], Minghao Xu[1], Mingze Lu[1], Guancheng Wang[1], Jingyuan Ma[1], Sitong Ding[1], Haoan Wu[1], Yu Zhang[1,**], Ming Ma[1,*]

[1] School of Biological Science and Medical Engineering, Southeast University, Nanjing, China

* e-mail:maming@seu.edu.cn

** e-mail:zhangyu@seu.edu.cn,

Table of contents

Supplementary figures

Supplementary Figure S1. TTC-stained brain slices from different groups.

Supplementary Figure S2. MALDI results from coronal sections of MCAo 2h.

Supplementary Figure S3. MALDI results from coronal sections of MCAo 2h reperfusion 1h, the only surgery group and MCAo 1h.

Supplementary Figure S4. The complete surgical procedure and corresponding stages of injury.

Supplementary Figure S5. Hierarchical Cluster Analysis (HCA) of metabolites labeled with KEGG tags (A to P).

Supplementary Figure S6. KEGG pathway analysis of brain injury related to metabolites from stereotactic probe insertion.

Supplementary Figure S7. KEGG pathway analysis of brain injury related to metabolites associated with subsequent metabolic changes caused by MCAo and surgery.

Supplementary Figure S8. Valine, Leucine, and Isoleucine Biosynthesis Pathway Metabolites Analysis

Supplementary Figure S9. Glutathione metabolism Pathway Metabolites Analysis

Supplementary Figure S10. Tyrosine metabolism Pathway Metabolites Analysis

Supplementary Figure S11. Pyrimidine metabolism Pathway Metabolites Analysis

Supplementary Figure S12. Parameter settings for each dimension in PLS-R (Partial Least Squares Regression).

Supplementary Figure S13. Unfolding of 10,529-dimensional metabolic features using i-PCA (incremental Principal Component Analysis).

Supplementary Figure S14. ROC curves for stage determination using the top 10 metabolites with the highest VIP (Variable Importance in Projection) values for each Component.

Supplementary Tables

Supplementary Table S1. Metabolic features upregulated in the metabolic abnormality area of the ischemic hemisphere.

Supplementary Table S2. Metabolic features downregulated in the metabolic abnormality area of the ischemic hemisphere.

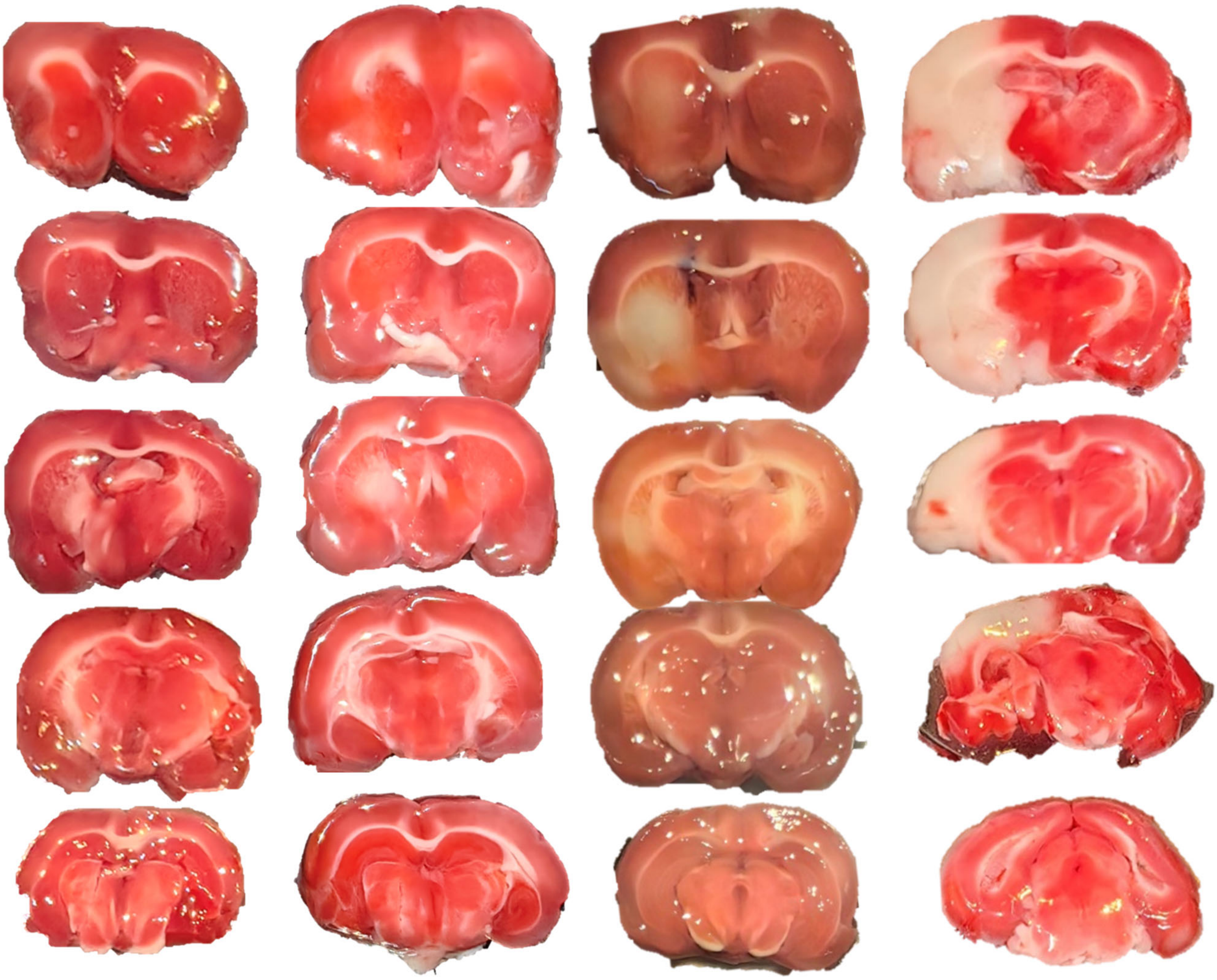

Supplementary Figure S1. TTC-stained brain slices from different groups. From left to right: the only surgery group (control group that only underwent surgical procedures without ischemia), MCAo 2h (middle cerebral artery occlusion for 2 hours), MCAo 2h reperfusion 1h (middle cerebral artery occlusion for 2 hours followed by 1 hour of reperfusion), and MCAo 24h (middle cerebral artery occlusion for 24 hours).

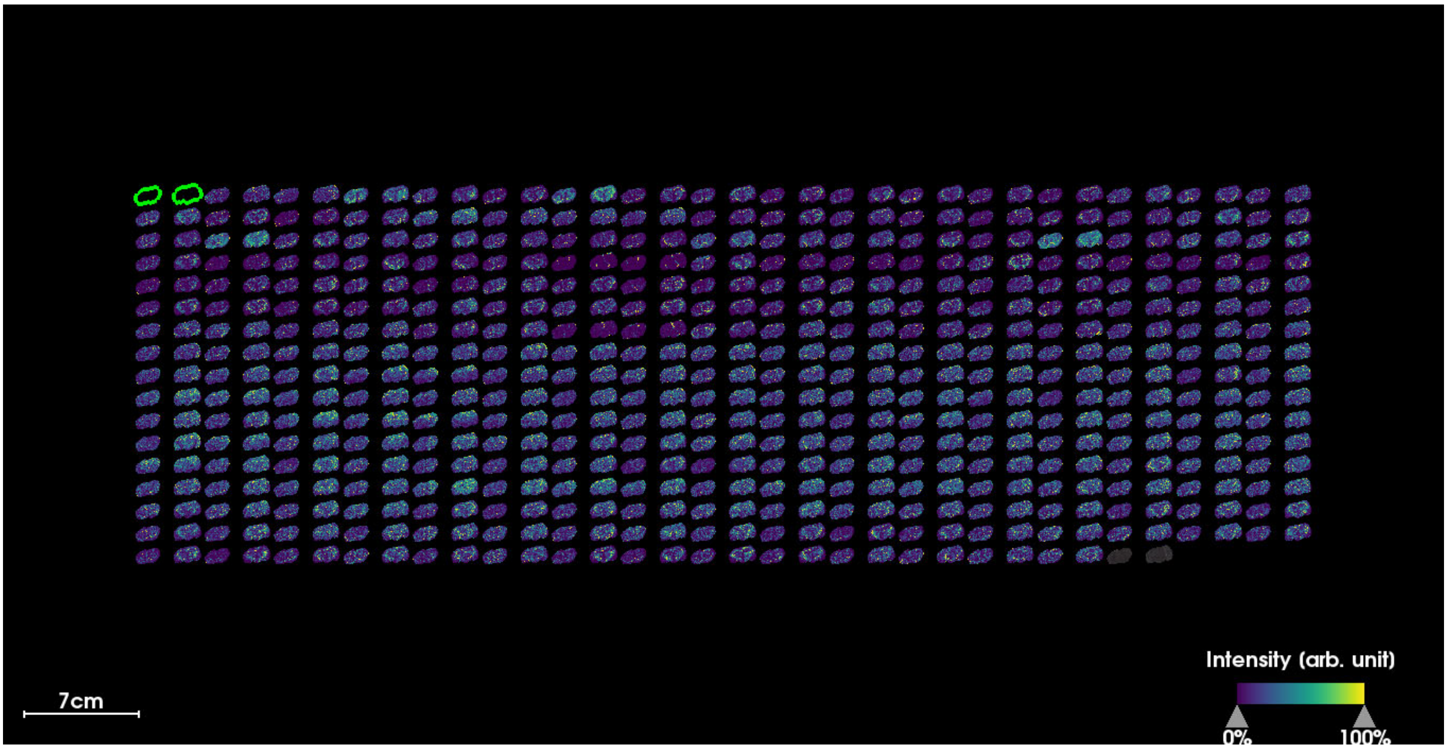

Supplementary Figure S2. MALDI results from coronal sections of brains subjected to 2 hours of ischemia. The sections include the hippocampus (left) and the striatum (right) regions. Each pair of slices represents the relative abundance of a specific metabolite across all pixels in these two regions.

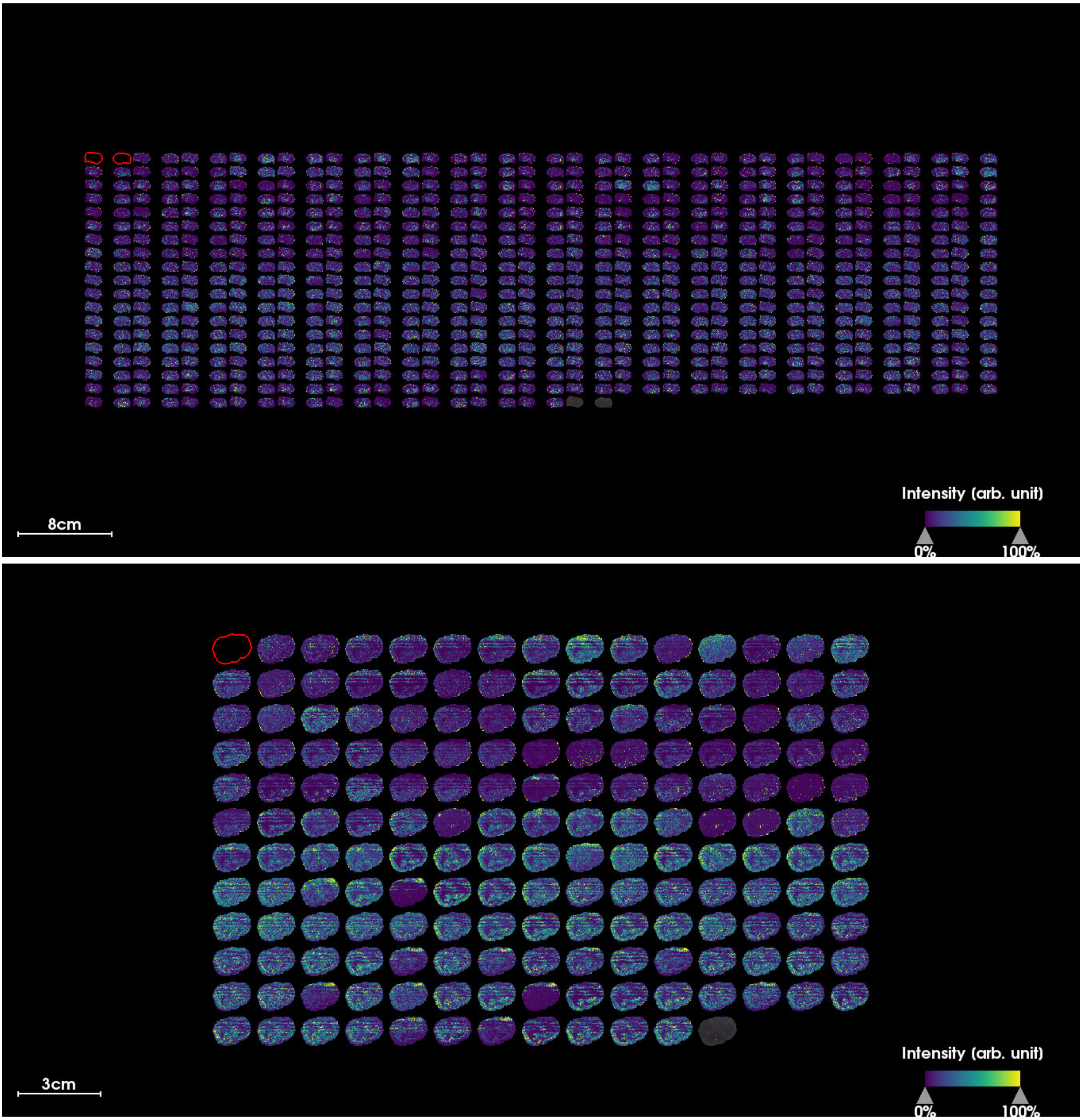

Supplementary Figure S3. MALDI results from coronal sections. The upper panels show results from MCAo 2h R 1h (left) and only surgery group (right). The lower panel shows results from MCAo 1h. Each pair of slices represents the relative abundance of a specific metabolite across all pixels in these two regions.

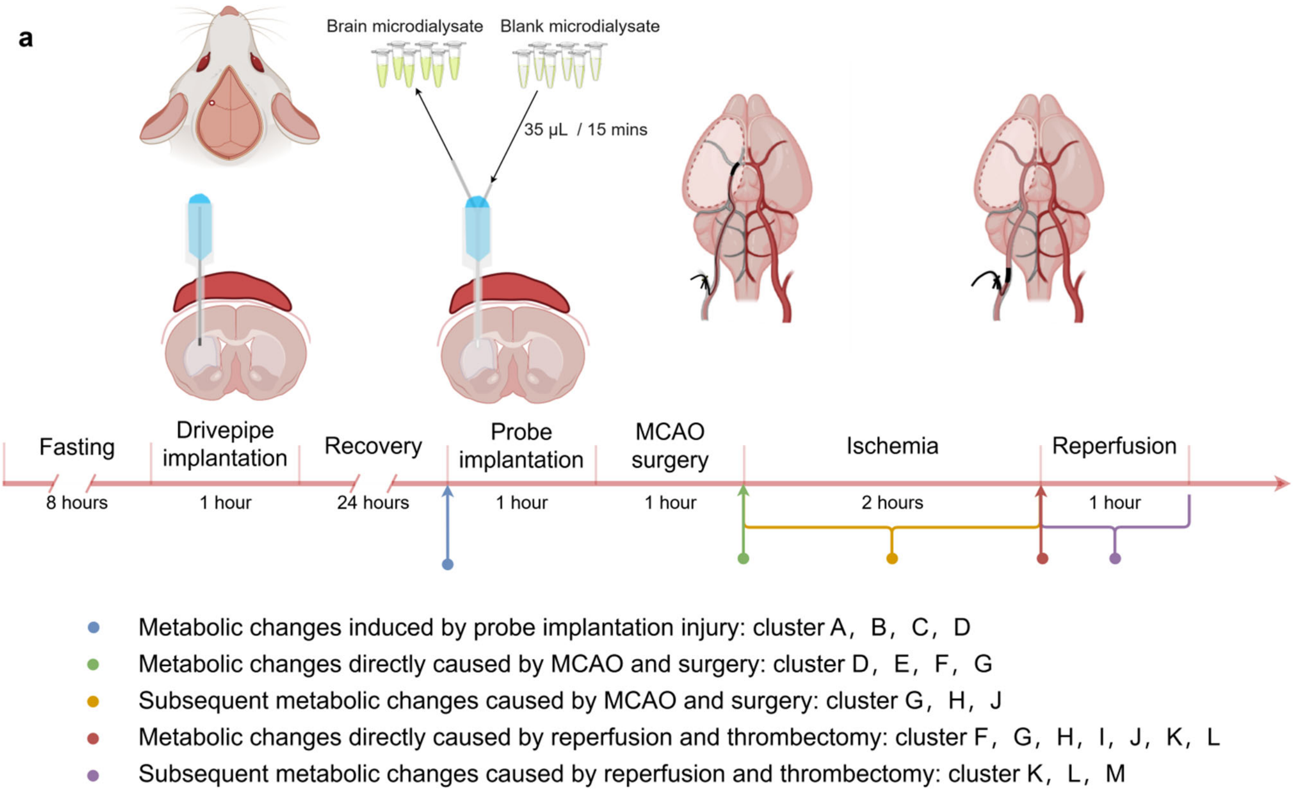

Supplementary Figure S4. The complete surgical procedure and corresponding stages of injury.

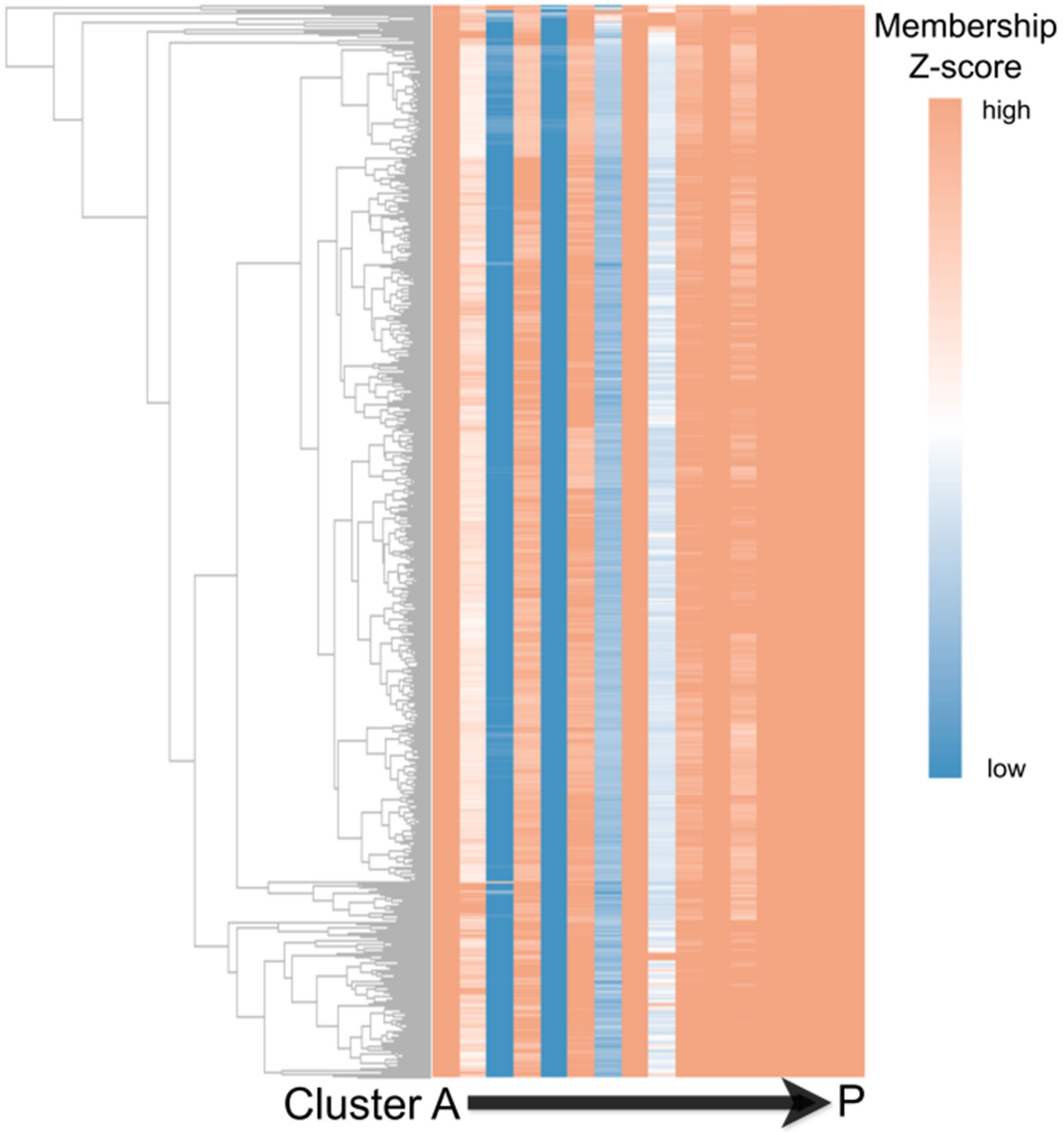

Supplementary Figure S5. Hierarchical Cluster Analysis (HCA) of metabolites labeled with KEGG tags (A to P).

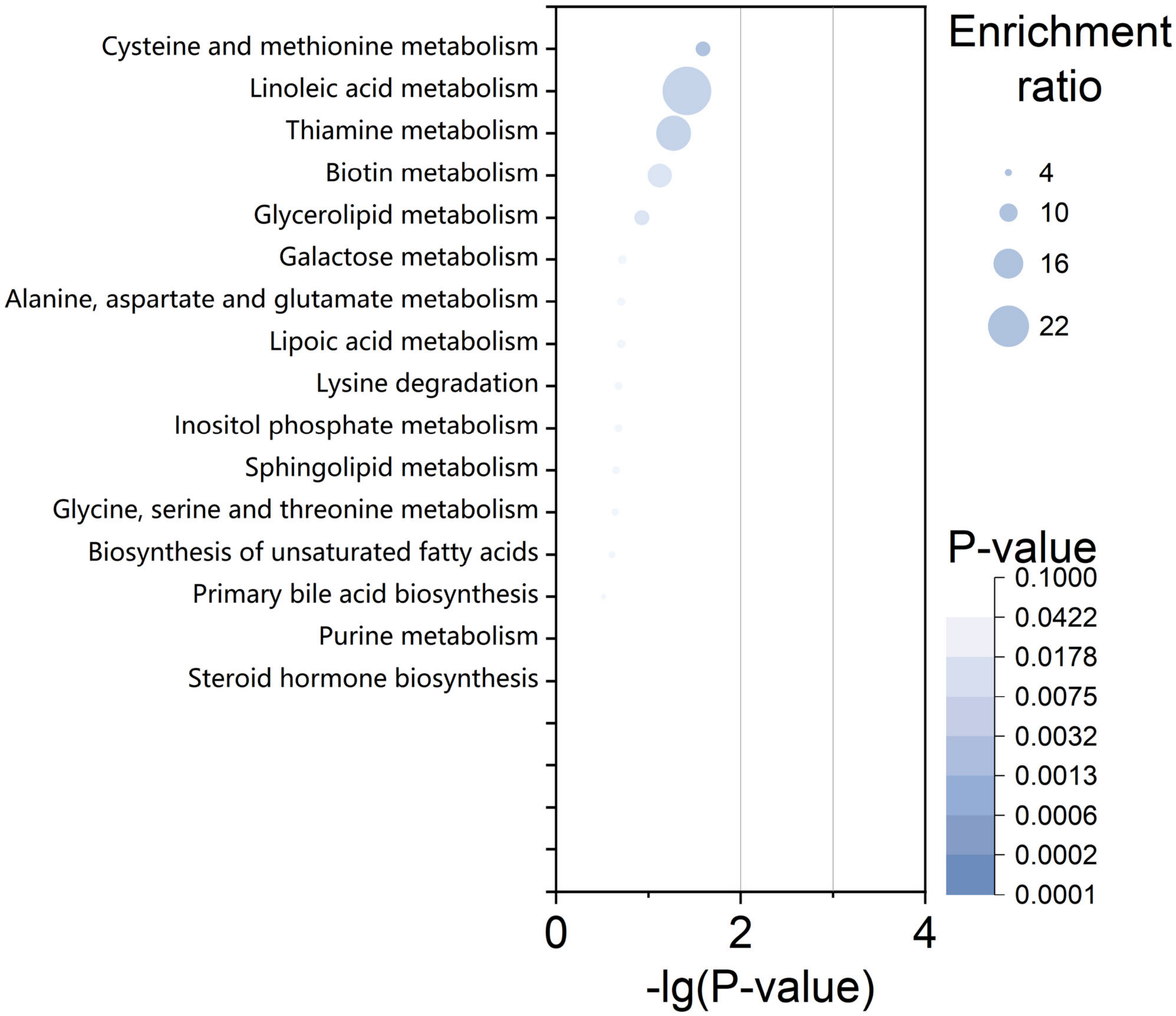

Supplementary Figure S6. KEGG pathway analysis of brain injury related to metabolites from stereotactic probe insertion.

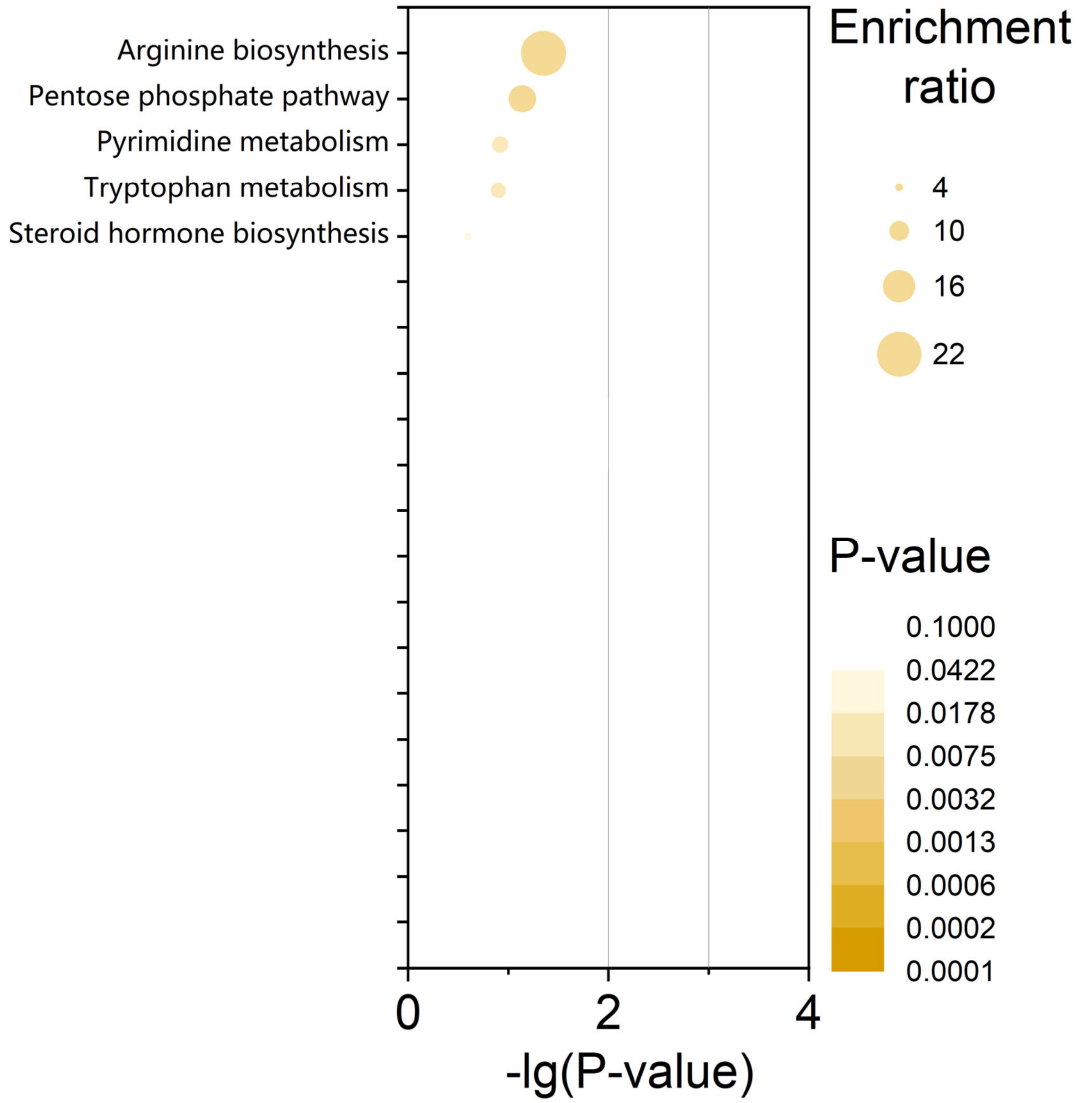

Supplementary Figure S7. KEGG pathway analysis of brain injury related to metabolites associated with subsequent metabolic changes caused by MCAo and surgery.

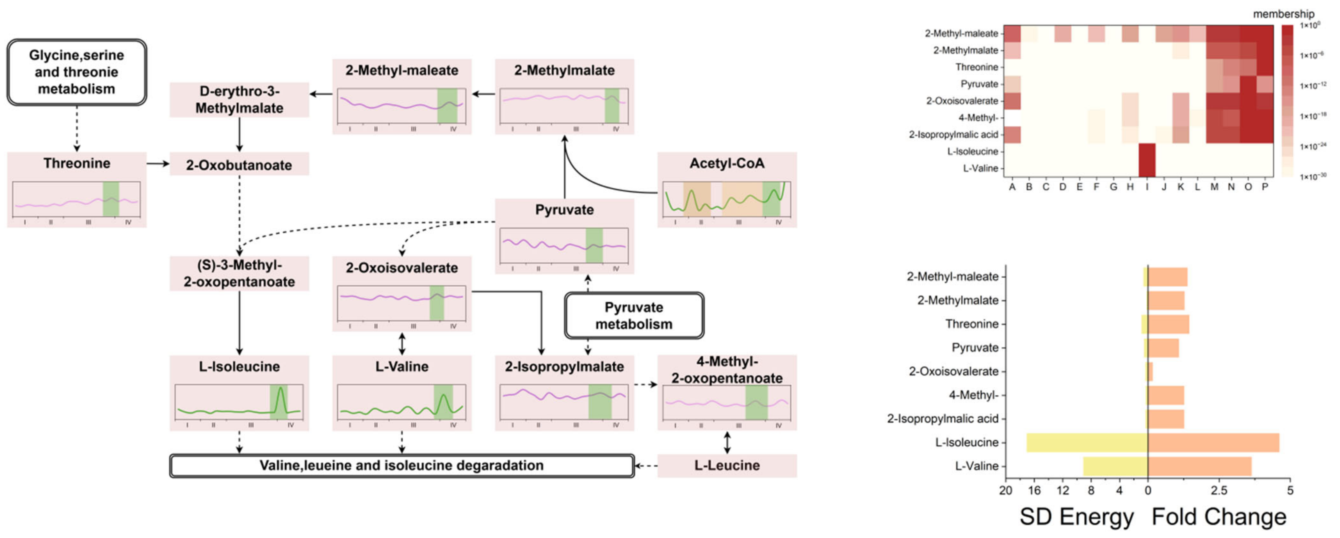

Supplementary Figure S8. Valine, Leucine, and Isoleucine Biosynthesis Pathway Metabolites Analysis. The right side shows the cluster-label mapping for arginine-biosynthesis metabolites and SD Energy (global temporal fluctuation) and key-time Fold Change (signature-period fold change).

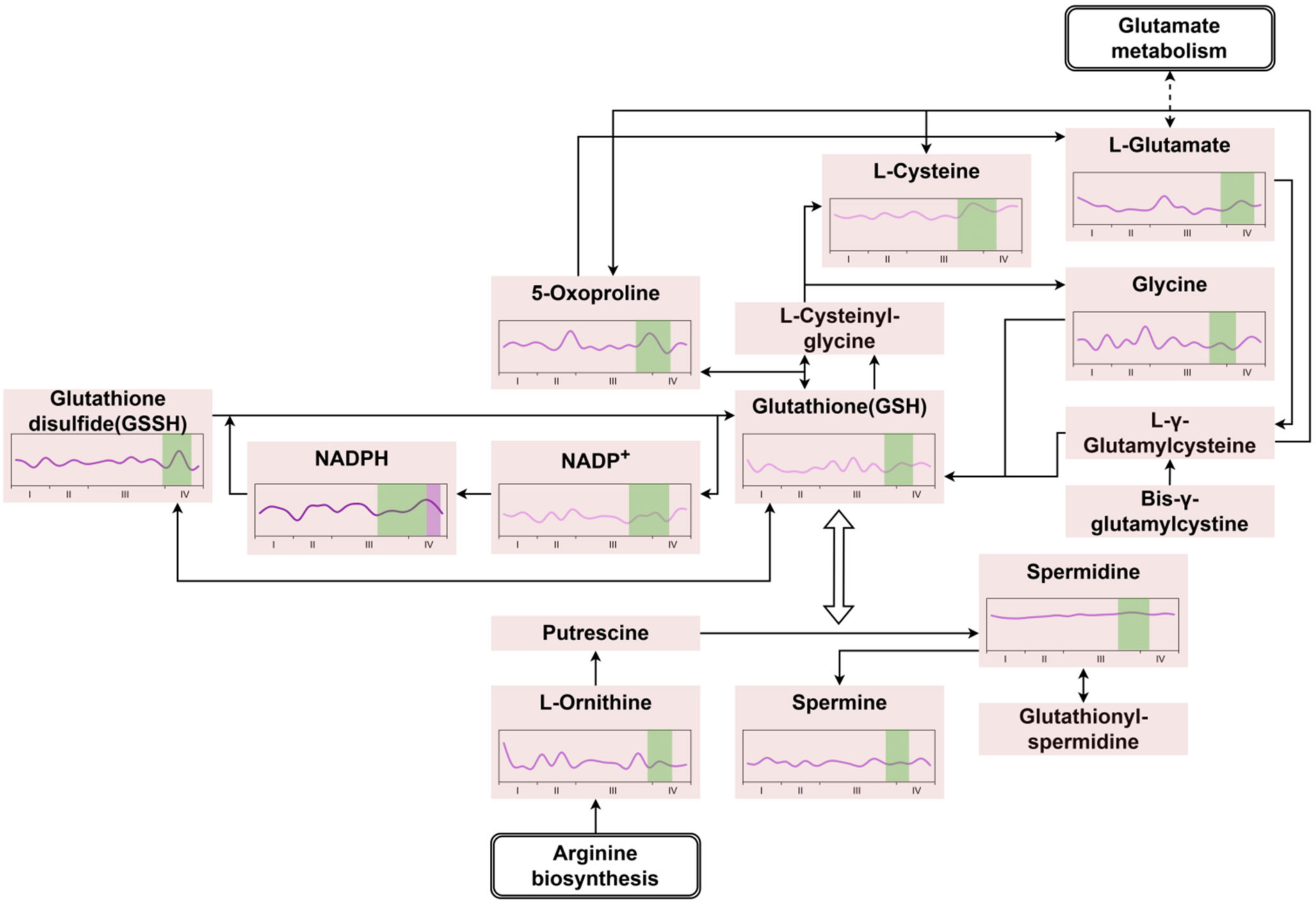

Supplementary Figure S9. Glutathione metabolism Pathway Metabolites Analysis.

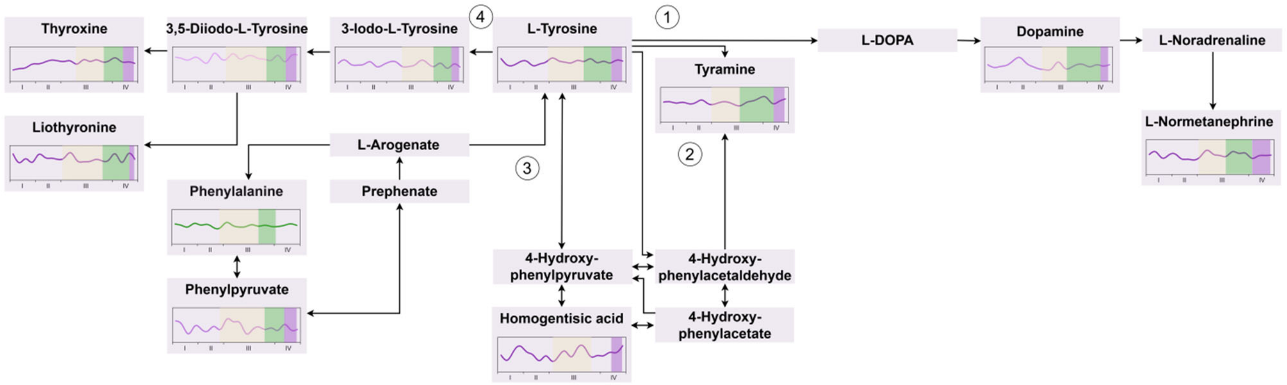

Supplementary Figure S10. Tyrosine metabolism Pathway Metabolites Analysis.

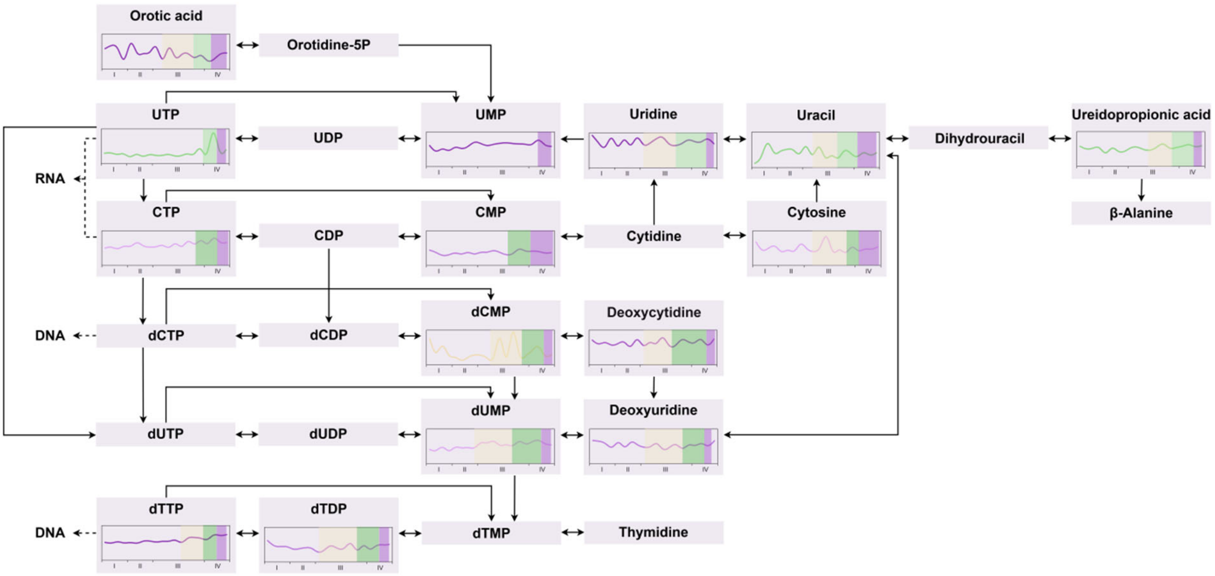

Supplementary Figure S11. Pyrimidine metabolism Pathway Metabolites Analysis.

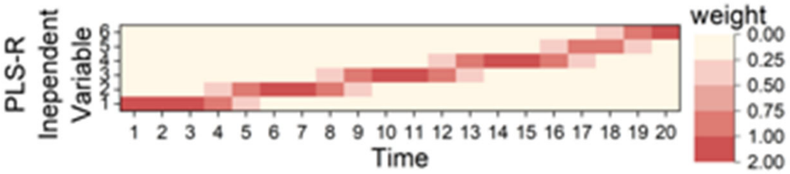

Supplementary Figure S12. Parameter settings for each dimension in PLS-R (Partial Least Squares Regression).

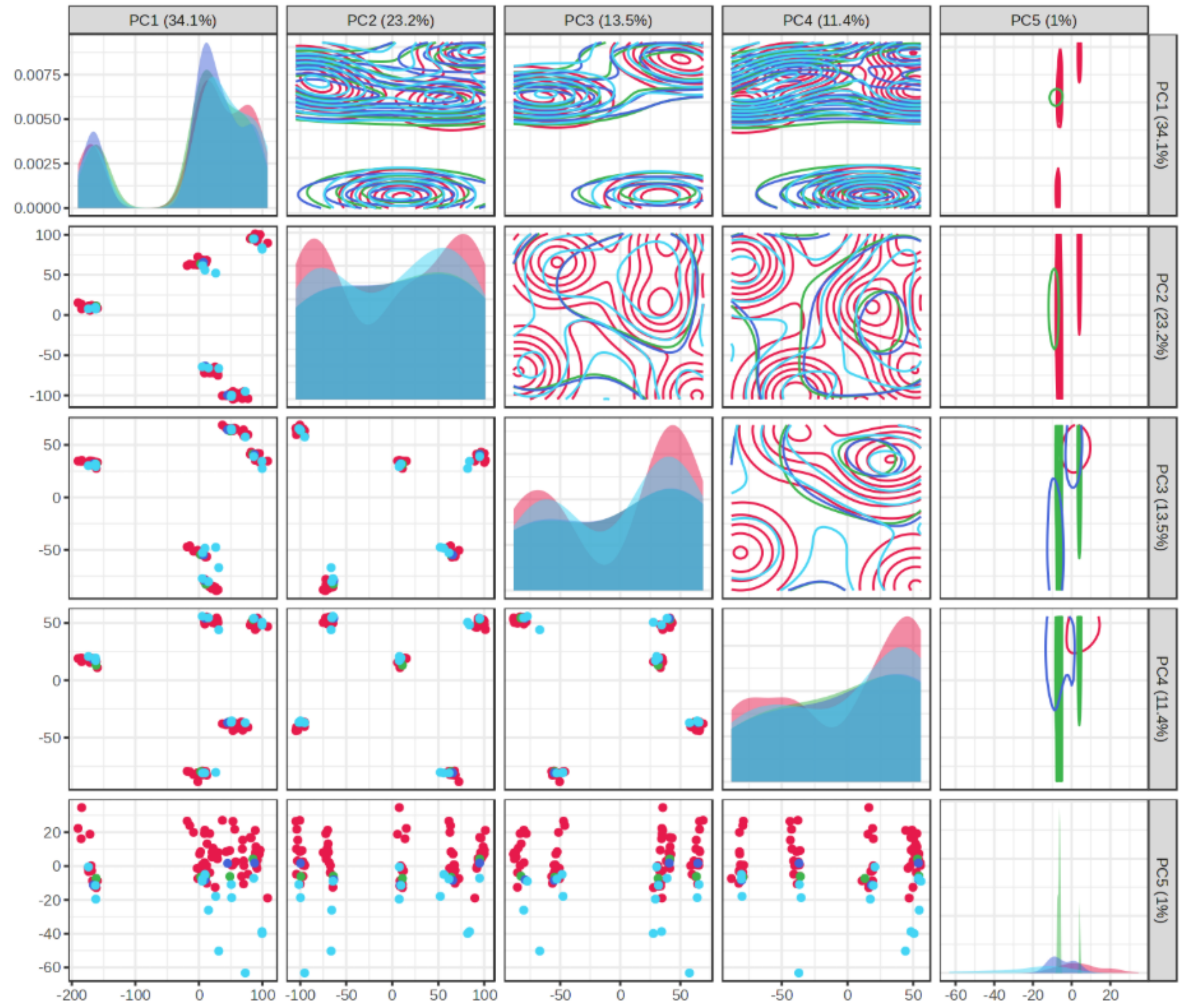

Supplementary Figure S13. Unfolding of 10,529-dimensional metabolic features using i-PCA (incremental Principal Component Analysis).

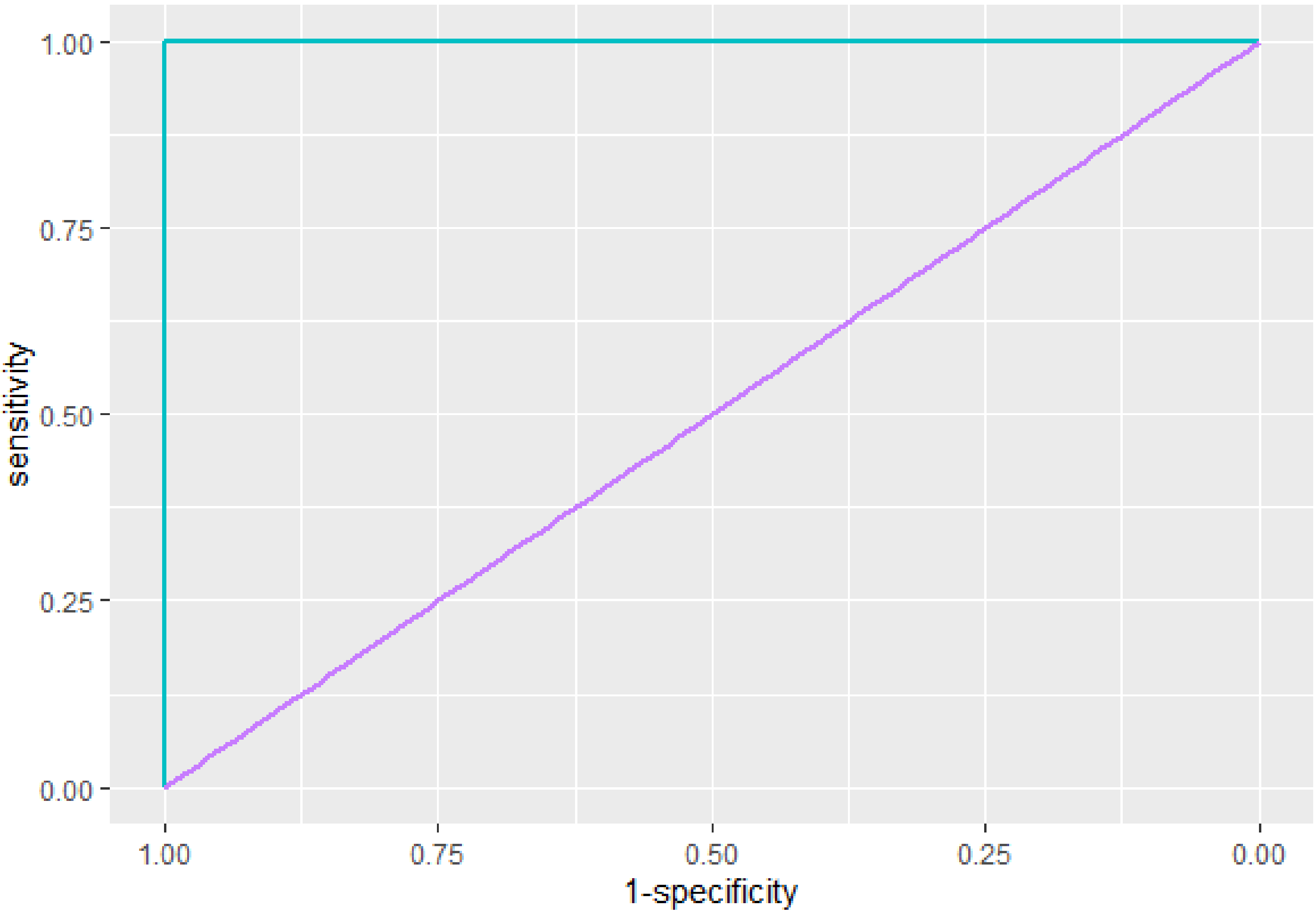

Supplementary Figure S14. ROC curves for stage determination using the top 10 metabolites with the highest VIP (Variable Importance in Projection) values for each Component.

Supplementary Table S1. Metabolic features upregulated in the metabolic abnormality area of the ischemic hemisphere.

m/z	ischemic	contralateral	Log_2(foldchange)
233.9662781	2.046206666	0.526092875	1.959562446
602.8223267	2.404783475	0.767940187	1.646841147
599.9434814	1.931884892	0.617659059	1.645126526
600.8431396	1.002707508	0.329044004	1.607548393
585.0092773	1.294538977	0.442912222	1.547345689
705.9221191	3.326488569	1.143447127	1.54061042
703.0432129	3.291926669	1.140561536	1.529187914
628.7322388	1.672621558	0.595472135	1.490005158
105.1365433	2.27686838	0.824319755	1.46577492
756.3024292	8.797500522	3.332517671	1.400481169
757.382019	3.660790464	1.429006907	1.35714231
586.0888672	0.969696688	0.392824847	1.303647372
104.0569687	25.94618761	11.14741611	1.218813229
732.9115601	1.140123463	0.502210547	1.182825828
198.1602478	1.148483135	0.512790439	1.163288404
706.8217773	1.085138635	0.48828169	1.152093785
707.9013672	0.673760139	0.306747982	1.135181226
731.8320313	1.758297083	0.81389284	1.111268088
784.371521	2.938135097	1.413620172	1.055506201
478.3109436	0.375576512	0.180885205	1.05403243
753.423584	2.316369721	1.134028513	1.030408629

Supplementary Table S2. Metabolic features downregulated in the metabolic abnormality area of the ischemic hemisphere.

m/z	ischemic	contralateral	Log_2(foldchange)
258.0768737	0.487007988	2.626746274	-2.431259511
299.1008606	0.044934573	0.227670687	-2.341050773
296.0420532	0.579683347	2.902847724	-2.324131947
349.1213379	0.07852278	0.350314218	-2.157466378
297.1216431	0.045706463	0.201549437	-2.140663677
339.2251892	0.078335772	0.31511134	-2.0081185
348.041748	0.65253396	2.590753006	-1.98924659
298.0212708	0.068112024	0.232763533	-1.772883632
423.9721375	0.157581199	0.51702429	-1.714136643
402.0206909	0.110902572	0.347319208	-1.646969373
266.5335693	0.191592682	0.562566894	-1.553982197
246.381424	0.069998839	0.201974101	-1.528767407
268.1529236	1.745155737	4.79030956	-1.456763101
172.9700775	0.166585027	0.434355625	-1.382617987
259.1564331	0.068910559	0.175051185	-1.344979859
492.8852539	0.399309147	0.984164648	-1.301393575
362.2562256	0.146864286	0.346389848	-1.237913036
228.5683746	0.126769503	0.297037459	-1.228437161
266.8934326	0.75751231	1.723778037	-1.186232778
269.0525818	0.405448493	0.914487012	-1.173444029
274.2705383	0.123222422	0.276454951	-1.165779608
866.239563	0.551610798	1.231893893	-1.159155395
280.0282898	0.12469057	0.276433074	-1.148577874
556.0405884	0.135116166	0.299178918	-1.146808218
136.0844727	0.676606016	1.482855068	-1.131989688
503.8609619	0.151476516	0.329025586	-1.119105634
152.0982208	0.142262446	0.293910688	-1.046822951
132.4858704	0.034099105	0.070360408	-1.045029985
319.2529907	0.073357571	0.150535095	-1.037082094
